# A Topical Microbicide Gel Formulation of CCR5 Antagonist Maraviroc Prevents HIV-1 Vaginal Transmission in Humanized RAG-hu Mice

**DOI:** 10.1371/journal.pone.0020209

**Published:** 2011-06-01

**Authors:** C. Preston Neff, Theresa Kurisu, Thomas Ndolo, Kami Fox, Ramesh Akkina

**Affiliations:** Department of Microbiology, Immunology and Pathology, Colorado State University, Fort Collins, Colorado, United States of America; University of California, San Francisco, United States of America

## Abstract

For prevention of HIV infection many currently licensed anti-HIV drugs and new ones in the pipeline show potential as topically applied microbicides. While macaque models have been the gold standard for in vivo microbicide testing, they are expensive and sufficient numbers are not available. Therefore, a small animal model that facilitates rapid evaluation of potential candidates for their preliminary efficacy is urgently needed in the microbicide field. We previously demonstrated that RAG-hu humanized mouse model permits HIV-1 mucosal transmission via both vaginal and rectal routes and that oral pre-exposure chemo-prophylactic strategies could be tested in this system. Here in these proof-of-concept studies, we extended this system for topical microbicide testing using HIV-1 as the challenge virus. Maraviroc, a clinically approved CCR5 inhibitor drug for HIV treatment, was formulated as a microbicide gel at 5 mM concentration in 2.2% hydroxyl ethyl cellulose. Female RAG-hu mice were challenged vaginally with HIV-1 an hour after intravaginal application of the maraviroc gel. Our results showed that maraviroc gel treated mice were fully protected against vaginal HIV-1 challenge in contrast to placebo gel treated mice which all became infected. These findings highlight the utility of the humanized mouse models for microbicide testing and, together with the recent data from macaque studies, suggest that maraviroc is a promising candidate for future microbicide clinical trials in the field.

## Introduction

More than a million women contract HIV infection annually through unprotected sex due to the lack of a simple self implementable preventive measure [1]. In this regard, an ideal vaginally applied anti-HIV microbicide will have a great impact in reducing the number of new cases [2], [3]. Such a microbicide will also empower women to protect themselves from HIV infected non-compliant partners. While a number of non-specific compounds proved to be ineffective in the early clinical trials, the recent significant success achieved with the RT inhibitor tenofovir demonstrated the merits of exploiting viral target specific compounds as potential HIV microbicides [3], [4]. There are currently more than 30 clinically licensed anti-HIV drugs and many more new ones are in the pipeline [5]. A number of these anti-HIV drugs show considerable promise as topically applied microbicides. However, for these to reach the clinical trial stage, it is necessary to conduct in vivo efficacy and safety testing in animal models.

Macaque models have long been used for HIV microbicide testing and are considered the gold standard because of many similarities with human genital tract anatomy and physiology [6]. Tenofovir is an example of a microbicide candidate that was initially evaluated in the monkey model [4]. However, due to the high cost and limited supply it is not feasible to solely rely on using macaques to conduct large scale initial screening of numerous compounds to identify a select number of new candidates. Another limitation with the macaque model is that it does not employ HIV-1 for challenge studies thus somewhat limiting its predictive value with regard to drugs that are specifically designed to be effective against HIV-1, but not SIV or SHIV viruses. Moreover, it is not possible to evaluate candidate microbicides against drug resistant and genetically divergent field HIV-1 viral strains. Based on these constraints, many potentially promising candidates will not be able to progress beyond in vitro testing. Therefore, a small animal model that can overcome some of the above limitations will greatly help speed-up the process of microbicide screening.

Humanized mouse models harboring HIV-1 susceptible human target cells can potentially be exploited for this purpose [7], [8]. The SCID-hu-PBL humanized mouse model prepared by passive transfer of human PBLs was utilized in this context in early microbicide evaluations [9], [10], [11]. However, the infection rate was found to be variable and therefore not fully reliable for microbicide testing due to low levels of repopulation by human cells in the vaginal mucosa [12]. To improve human cell engraftment, more recent humanized mouse models incorporated transplantation of human hematopoietic stem cells (CD34^+^ cells) into immunodeficient mice with much compromised innate immunity [7], [8], [13]. Mouse strains such as NOD/SCIDγc^−/−^ and Rag2^−/−^γc^−/−^ allowed superior human cell engraftment levels resulting in a more robust multilineage human hematopoiesis and engraftment of primary and secondary lymphoid organs [8], [13]. Another model, the BLT mouse model, is created by an improvement of the conventional SCID-hu mouse model which involves transplantation of thymic and liver tissues under the kidney capsule followed by injection with autologous human CD34^+^ cells [14]. Both these humanized mouse models have been shown to support intestinal, vaginal and rectal mucosal tissue engraftment by human HIV-1 target cells such as CD4+ T cells and macrophages [15], [16], [17]. A number of groups including ours have demonstrated the utility of these humanized mice as improved models for HIV-1 infection and CD4 T cell depletion [18], [19], [20], [21], [22], [23]. Importantly, these models also permit HIV-1 mucosal transmission via both vaginal and rectal routes [15], [24] thus making it possible to experimentally evaluate novel prevention strategies of HIV-1 sexual transmission. Indeed, in a pre-exposure prophylactic approach (PrEP), systemic administration of TDF (tenofovir) and FTC (emtricitabine) was found to prevent HIV-1 vaginal transmission using the BLT mouse model [24], [25]. In a similar approach, using the RAG-hu humanized mouse model, we recently showed that oral administration of two clinically approved new generation compounds namely, the integrase inhibitor raltegravir and a CCR5 inhibitor maraviroc confers protection against HIV-1 vaginal challenge [26].

However, successful testing of topically administered microbicide compounds for prevention of HIV-1 sexual transmission has thus far not been reported using either of these mouse models. The current list of potential anti-HIV microbicide candidates is dominated by post-entry viral inhibitors such as tenofovir and emtricitabine [4], [27]. As pointed out above, tenofovir has already been tested in human clinical trials and showed partial protection in the field [4]. For an ideal microbicide, it is even more desirable to prevent viral entry which is the first step in viral infection. In this regard, the clinically approved CCR5 inhibitor maraviroc holds great promise and is being considered as a potential anti-HIV microbicide, and indeed recent macaque studies demonstrated that it can protect against a SHIV virus vaginal challenge [28]. Here we show that vaginal application of a gel formulation of maraviroc confers full protection against HIV-1 vaginal challenge in RAG-hu mice thus also validating a new small animal model for future HIV microbicide testing.

## Materials and Methods

### Preparation of humanized Rag2^−/−^γc^−/−^ mice (RAG-hu mice)

Humanized BALB/c-Rag2^−/−^γc^−/−^ and BALB/c-Rag1^−/−^γc^−/−^ (RAG-hu) mice were generated by transplanting with human fetal liver-derived CD34^+^ hematopoietic progenitor cells as we previously described [15], [20]. Mice were maintained at the Colorado State University Painter Animal Center. These studies have been reviewed and specifically approved by the CSU Institutional Animal Care and Use Committee (Protocol 09-085A). Briefly, newborn mice were conditioned by irradiating at 350 rads and then injected intrahepatically with 0.5−1×10^6^ human CD34^+^ cells. Mice were screened for human cell engraftment at 10–12 weeks post-reconstitution. Peripheral blood was collected by tail bleed and red blood cells were lysed by using the Whole Blood Erythrocyte Lysing Kit (R&D Systems, Minneapolis, MN). The white blood cell fraction was stained with antibodies against the human pan-leukocyte marker CD45 (Caltag) and FACS analyzed to determine the levels of human cell engraftment as we previously described [20]. Female mice prepared with different donor CD34+ cells with over 60% engraftment (Table 1) were randomly picked for vaginal infections as detailed below. Mice used in these experiments were not monitored for estrus.

**Table 1:** Summary of human cell engraftment levels in humanized mice*.

Uninfected Control			Placebo		
Mouse	Gender	%Engraftment	Mouse	Gender	%Engraftment
889	Female	75.9	924	Female	63.7
890	Female	81.5	925	Female	66.4
			926	Female	67.0
			J848	Female	92.5
			J849	Female	82.5
			J850	Female	93.8

Maraviroc Treated					
Mouse	Gender	%Engraftment			
912	Female	85.5			
913	Female	88.8			
J830	Female	94.0			
J831	Female	80.0			
J832	Female	90.6			
J833	Female	94.3			
J834	Female	83.2			

*Peripheral blood was collected from human CD34 cell reconstituted RAG-hu mice (BALB/c-Rag2^−/−^γc^−/−^ or BALB/c-Rag1^−/−^γc^−/−^, the prefix J is indicative of RAG1) at 10–12 weeks post engraftment. White blood cell fraction was stained with human CD45 FITC conjugated antibody and analyzed by FACS to confirm human cell engraftment prior to maraviroc or placebo gel treatments and vaginal HIV-1 challenges.

### Vaginal application of maraviroc gel and HIV-1 challenge by vaginal route

Female RAG-hu mice were topically administered with either a placebo gel (6 mice) or maraviroc gel (7 mice) an hour before viral challenge. Clinical formulation of Maraviroc in a 150 mg tablet form ((Selzentry, Pfizer Labs) was ground and freshly dissolved in phosphate-buffered saline, sterile-filtered and adjusted to a final concentration of 4 mg/mL (7.8 mM). A 3.4% gel preparation of hydroxyl-ethyl cellulose (HEC) was added to the dissolved drug to achieve a final concentration of 5 mM maraviroc in 2.2% HEC gel. A 25 µl volume of the gel formulation was carefully applied in to the vaginal vault of mice. Control mice received 2.2% HEC placebo gel. An hour post-gel application, mice were challenged vaginally with HIV-1 BaL (3000 TCID) in a 25 µl volume. The gel and viral inoculums were applied by using the bulbous end (1.25 mm in diameter) of a newborn mouse straight 24 gauge gavage needle (purchased from VWR, made by POPPER, N.Y) to assure no abrasions and tearing. Mice were anesthetized by isoflurane inhalation for 7–10 minutes during the procedures. Anesthetized mice were held in an inverted position for four minutes post-inoculation to allow virus to adsorb and to prevent immediate discharge of virus as described previously [15], [26]. Animals were observed daily and blood samples collected on a weekly basis to assess plasma viremia and CD4 T cell counts.

### Measurement of viral loads

Viral infection and viral loads were assessed by Q-RT-PCR. RNA was extracted from 25–50 ul of EDTA-treated plasma using the QIAamp Viral RNA kit (Qiagen, Valencia, CA). Q-PCR was performed by using a primer set specific for the HIV-1 LTR sequence and a corresponding LTR specific probe as described previously [15], [29]. To detect integrated form of the virus, cellular DNA was extracted using QIAmp DNA Blood kit (Qiagen, Valencia, CA) and subjected to q-RT-PCR by the iQ SYBR Green Supermix (Bio-Rad, Hercules, CA) to determine proviral loads.

### Flow cytometry

Mice were monitored to measure the levels of CD4 T cells in the peripheral blood. Whole blood was collected and red blood cells lysed as reported previously [20], [29]. Peripheral blood cells were stained for hCD3-PE and hCD4-PECy5 (Caltag) markers and analyzed using a Coulter EPICS XL-MCL FACS analyzer (Beckman Coulter, Fullerton, CA). CD4^+^ T cell levels were calculated as a ratio of the entire CD3 population (CD4^+^CD3^+^:CD4^−^CD3^+^). All mice were analyzed prior to infection to establish baseline CD4^+^ T cell ratios.

## Results

### Vaginal application of maraviroc gel protects humanized mice against HIV-1 vaginal challenge

**Figure 1:**
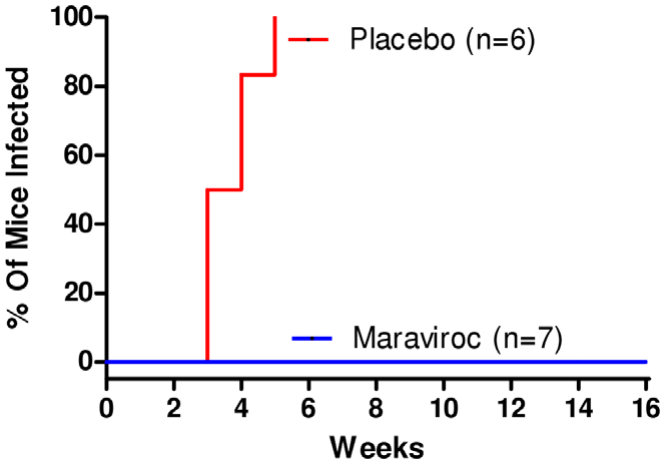
Vaginal application of maraviroc gel protects humanized mice against vaginal HIV-1 challenge. RAG-hu mice were challenged by vaginal route one hour after vaginal administration of maraviroc as described in Methods. Blood was collected weekly from infected mice and the status of HIV-1 infection was determined by Q-RT-PCR. Kaplan-Meier plots of time course of appearance of viremia in drug treated versus non-treated virus challenged mice.

**Figure 2:**
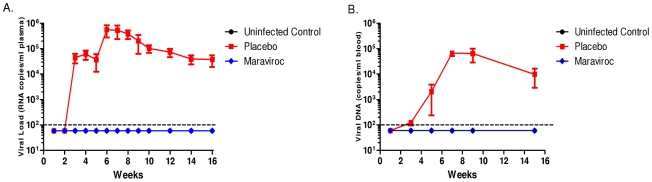
RNA and DNA viral loads in mice administered with maraviroc. RAG-hu mice were challenged by vaginal route after an hour after vaginal application of maraviroc as described in Methods. Blood was collected weekly. Viral RNA was extracted from the plasma fraction and DNA was extracted from the cellular fraction. Viral RNA and DNA loads were determined by Q-PCR as described in methods. The dotted lines represent limits of PCR detection. A. RNA viral loads B. DNA viral loads.

We have previously established that RAG-hu humanize mice permit mucosal HIV-1 infection by both vaginal and rectal routes [15] and have recently shown that oral administration of anti-retrovirals, maraviroc and raltegravir protect these mice against HIIV-1 vaginal challenge [26]. Here we evaluated maraviroc as a topically applied microbicide gel to prevent HIV-1 sexual transmission. To assess efficacy, maraviroc was administered vaginally one hour prior to vaginal viral challenge to mimic a coital dependant context. Mouse plasma and blood cellular fractions were analyzed by Q-PCR weekly and bi-weekly respectively to ascertain the HIV-1 infection status. Our results showed that the placebo-gel administered and HIV-1 challenged mice started becoming virus positive by the third week with all of them infected by the 5th week post challenge (Fig. 1). Persistent viremia in plasma and proviral loads in the cellular fractions were observed throughout the evaluation period with viral loads reaching up to 10^6^ RNA copies/ml (Fig. 2A) and proviral DNA copies at 10^5^ DNA copies/ml (Fig. 2B). In contrast, none of the maraviroc treated mice became infected (Fig. 1). No evidence of infection was seen throughout the 16 week observation period as evaluated by either RNA or DNA PCR (Fig. 2A and B). These data taken together suggest that vaginal administration of maraviroc fully protects mice against HIV-1 vaginal challenge.

### CD4 T cell loss in placebo-gel administered mice compared to maraviroc-gel protected mice

**Figure 3:**
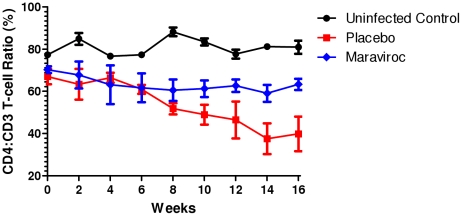
CD4 T cell decline in non-treated vaginally challenged mice in contrast to mice protected with maraviroc gel. Levels of CD4 T cells were monitored on a weekly by FACS basis to determine their decline in treated versus non-treated mice. Baseline values for each of the mice were established prior to infection as described in Methods.

A typical finding in HIV-1 infected humanized mice is a gradual CD4 T cell loss as seen in the human. Although the PCR data demonstrated no viral infection in maraviroc treated mice, we further evaluated the virus challenged mice for any evidence of CD T cell loss to confirm lack of any HIV associated pathology [20], [29]. Peripheral blood was collected bi-weekly and subjected to FACS analysis. To establish a baseline, CD4 T cell levels were measured prior to viral challenge for each of the experimental mice and later compared to the levels determined post-viral challenge. While there was a clear pattern of CD4 T cell decline in placebo-gel treated and viral challenged mice, their levels were stable in mice receiving maraviroc gel (Fig. 3) further validating the prevention of HIV-1 transmission in these mice.

## Discussion

With the dearth of a protective vaccine in the immediate future, deployment of an effective vaginally applied anti-HIV microbicide would greatly aid in stemming the HIV epidemic [3]. To reach this goal, new viral specific anti-HIV compounds need to be tested as topical microbicides using a suitable in vivo system. In these proof-of-concept studies, we have shown that a topical microbicide gel formulation of the CCR5 inhibitor drug maraviroc fully protects humanized mice from HIV-1 challenge via vaginal route which is the predominant mode of viral transmission. While a recent study had shown the efficacy of maraviroc in preventing vaginal infection in rhesus macaques by a CCR-5 using SHIV-162P3 hybrid virus [28], our present results demonstrated the efficacy of the drug against HIV-1 in a human target cell context in humanized mice. In addition to supporting the results of the macaque studies, our findings extended them to the primary culprit HIV-1 itself against which the drug was originally designed and intended thus providing a more direct evidence.

Due to its capacity as a CCR5 antagonist, the small molecular drug maraviroc inhibits the binding of native physiological ligands of CCR5, namely CCL3 (also known as MIP-1a), CCL4 (MIP1-b) and CCL5 (RANTES) [30]. It is shown to have sustained antiviral activity due its long lasting physical and functional occupancy of CCR5 receptor. It has potent effect against all R5 tropic viruses representing various viral clades, and is also shown to be effective against a wide array of drug resistant viruses [31]. A major advantage with drugs such as maraviroc compared to RT inhibitors is that the first step in infection, viral entry, is inhibited thus providing an up-front protection.

To simulate the use of the microbicide in coital context, maraviroc gel was applied vaginally one hour before HIV-1 R5 viral challenge. The 5 mM maraviroc concentration we used is close to the dose (6 mM) found to be most protective in monkey studies using a similar 2.2% HEC carrier gel formulation [28]. However, in contrast to the above studies, it was not necessary to subject the humanized mice to progesterone hormonal (Depo-Provera) treatment to induce vaginal thinning to facilitate efficient vaginal infection. While all the placebo-gel treated, vaginally HIV-1 challenged mice (n = 6) became infected within five weeks, none of the maraviroc treated mice (n = 7) showed any evidence of infection by either RT-PCR or DNA-PCR throughout the sixteen week observation period. Thus protection conferred by maraviroc gel against HIV-1 vaginal challenge is highly significant (p value 0.0006, Fisher's exact test). In addition to viral detection, we also looked for any evidence of helper CD4 T cell loss which is a typical feature of HIV-1 infection. A trend of declining CD4 T cell counts was noticed as expected in placebo-gel treated mice in contrast to those receiving maraviroc (Fig. 3). These data taken together showed that maraviroc fully protected mice from vaginal HIV-1 challenge.

The above promising data together with those using SHIV viral challenges in macaques strongly suggest maraviroc as an attractive candidate for further development as a topically administered anti-HIV microbicide. Since it is not yet widely used in other parts of the world where HIV prevalence is high, it will have a good resistance barrier. However it should be noted that maraviroc will not protect against X4 tropic and/or dual tropic viruses. With regard to the drugs that are in clinical trials or nearing that stage, the HIV microbicides arena is currently focused on only a few HIV-1 specific compounds such as RT inhibitors tenofovir and emtricitabine [27]. While the partial protection afforded by tenofovir gel is encouraging, it is by no means adequate to be deployed as a sole candidate to protect the large at-risk population [4]. Therefore, it is necessary that many new compounds be tested to identify candidates with a good chance for success. Furthermore, it is generally understood that a single drug would not be adequate to achieve broad efficacy and therefore a combinations of drugs need to be tested [2], [32], [33]. Such testing in a primate model will be cost-prohibitive. In this regard, humanized mice are likely to fill the gap for deriving preliminary data and in laying the ground work for subsequent macaque studies. Since HIV-1 itself can be used as a challenge virus, various drug resistant mutants that exist in the field can also be evaluated against different combinations of potential promising compounds.

While both RAG-hu and BLT mouse models have been successfully used to demonstrate the efficacy of systemically administered anti-HIV drugs for pre-exposure prophylaxis [25], [26], our present results have extended the utility of RAG-hu mouse model for topical microbicide testing as well. Both these models are likely to play an important role in the development of new PrEP strategies that encompass systemic and/or topical use of anti-HIV drugs. In this context, the RAG-hu mouse model offers some practical advantages over BLT mice. First, its preparation is not technically intensive as no surgical procedure is required to implant thymic and liver tissues under the kidney capsule as is necessary to generate BLT mice. Second, RAG-hu mice have a longer life span compared to BLT mice (NOD/SCID mice used to prepare BLT mice experience a high incidence of lymphomas), thus permitting long-term studies. For example, effect of microbicides on the mucosal membranes during long-term application can be evaluated. Third, for any large scale testing, an adequate cohort of humanized mice is needed. In this regard, greater numbers of RAG-hu mice can be generated per human tissue donor compared to BLT mice. For example, depending on available tissue quantity and CD34+ cell yield, 30–60 mice can be generated.

In summary, we have shown that a maraviroc gel can prevent HIV-1 vaginal transmission. These data also helped validate the utility of humanized mice for testing topical microbicides. Now it is possible to conduct large scale in vivo preliminary screenings of a large number of potential microbicide candidates in a cost effective manner. In addition, a number of important questions regarding protection against cell associated virus, influence of estrus and low dose repeated viral challenges on the microbicide efficacy can be evaluated in the future.
